# *In silico* characterization of a putative ORF-MAP1138c of *Mycobacterium avium* subspecies *paratuberculosis* (MAP) with its implications in virulence

**DOI:** 10.1186/1471-2164-15-S2-P14

**Published:** 2014-04-02

**Authors:** Syed A Hassan, Seyed E Hasnain, Sami M Halawani

**Affiliations:** 1 Faculty of Computing and Information Technology Rabigh, King Abdulaziz University, PO Box 344, Rabigh 21911, Kingdom of Saudi Arabia; 2 Kusuma School of Biological Sciences, Indian Institute of Technology, Hauz Khas, New Delhi 110016, India; 3 Faculty of Computing and Information Technology Jeddah, P.O. Box 80200, Zip Code 21589, King Abdulaziz University, Kingdom of Saudi Arabia

## Background

Johne’s disease is a chronic mycobacterial infection of the small intestine affecting ruminants worldwide. It is estimated that over 50% of the European Union (EU) dairy holdings is infected [1]. The causal agent is *Mycobacterium avium* subspecies *paratuberculosis* (MAP), a slow-growing, acid-fast bacterium. It is a part of the *Mycobacterium avium* complex (MAC), which also comprises of opportunistic pathogens of humans, as well as innocuous, environmental bacteria [2]. MAP generally interacts with macrophages via different types of receptors, including Toll-like receptors (TLRs) [3,4]. It has been demonstrated of late that H37Rv1411c (LprG) enhances the recognition of triacylated *Mycobacterium tuberculosis* glycolipids by TLR2 and thereby restraining the expression of MHC-II molecules and processing of antigen and presentation of MHC restricted antigens by macrophages in a TLR2-dependent manner [5,6]. However, little is known about how *M. paratuberculosis* evades and resists this active CD4^+^ T-cell response and survives and infects other macrophages, a hallmark of mycobacterial infections. In this context, the identification of antigenic proteins is useful in understanding the immune evasion mechanism of MAP within host macrophages.

## Materials and methods

In this study, a comparative proteomic analysis of an orthologous putative gene MAP1138c or LprG and H37Rv1411c (LprG) was done using online bioinformatics tools namely ProtPram and SignalP 4.1, Phosphor 2.1, ProtScan and Hydropathy plot. The theoretical 3D structure of MAP1138c was generated using SWISS MODEL server using H37Rv1411c (LprG) as template. The secondary and super secondary structures of MAP1138c protein were identified and analyzed using PROMOTIF. The theoretical 3D structure generated was further assessed for its reliability using QMEAN, ANOLEA and GROMOS structure assessment tools of SWISS MODEL server.

## Results

Gapped BLAST of MAP1138c identified a homolog having sequence identity match of 70 % and positive match of 83%, respectively with crystal structure of H37Rv1411c (LprG). Proteomics analysis reveals that MAP1138c is a prolipoprotein and is translocated to the cell membrane using Tat pathway and probably the mature protein is secreted out after cleavage by SPase I peptidases. The ProtPram analysis shows similarity in physiochemical parameters namely Grand average of hydropathicity, aliphatic index and instability index for both H37Rv1411c (LprG) and MAP1138c proteins (Table 1).

**Table 1:** A comparative study of the physiochemical parameters of MAP1138c and H37Rv1411c (LprG) (*GRAVY (-ve) = hydrophilic nature and (+ve) = hydrophobic nature

*Protein*	*Molecular weight (kD)*	*Amino acid composition*	*Instability Index ( > 40 = unstable)*	*Aliphatic Index*	Grand Average of Hydropathicity (GRAVY)*
MAP1138c	24.72	238	28.31(Stable)	89.33	-0.105
H37Rv1411c (LprG)	24.54	236	16.11(Stable)	86.86	-0.127

**Figure 1:**
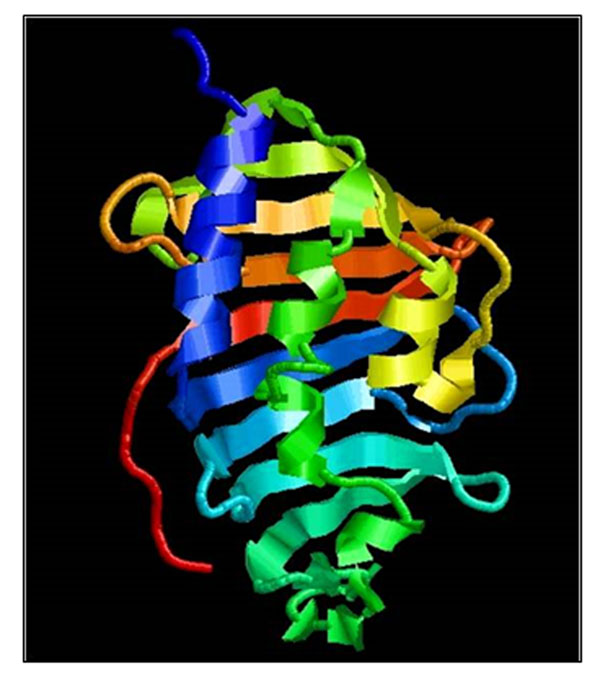
3D model of MAP1138c protein generated by SWISS-MODEL server.

The comparative hydropathy plot revealed that both MAP1138c and H37Rv-LprG proteins are highly antigenic and hydrophilic by nature. ProtScan domain analysis show the presence of DUF1396 domain specifies that MAP1138c belongs to LppX/LprAFG lipoprotein family of Mycobacterium species and possibly play an important role in the evasion of immune response within host macrophages. The 3D model of MAP1138c, generated by SWISS-MODEL server (Figure 1), shares secondary structure with H37Rv1411c (LprG), as brought forth by PROMOTIF analysis.

**Figure 2:**
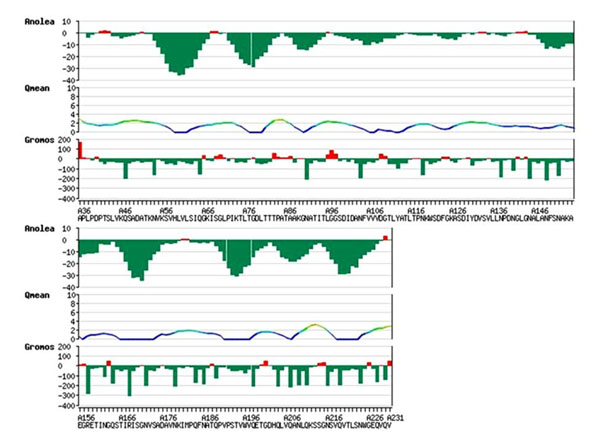
Graphical representation of ANOLEA, QMEAN and GROMOS analysis of MAP1138c protein model.

The hydrophobic residues lining the central cavity and portal of the protein model supports our hypothesis that like H37Rv1411c-LprG, MAP1138c can bind lipids or lipopolysaccharides (TLR2 agonists) in the cavity and initiates a TLR2 mediated immune evasion. The analysis of QMEAN , ANOLEA and GROMOS structure assessment tools reveal that the global and local properties of MAP1138c protein model generated by automated SWISS-MODEL server using H37Rv1411c (LprG) as template is reliable and can be used for further analysis (Figure 2).

## Conclusions

Through our comprehensive *in silico* analysis of MAP1138c, we identified several key aspects fundamentally important to propose a function for this putative protein. These studies reflect upon the possible role of MAP1138c in inhibiting MHC-II Ag processing leading to reduced recognition of infected macrophages by CD4^+^ T cells. This may be an important mechanism for immune evasion during persistent *M. paratuberculosis* infection in ruminants and humans.
